# ﻿Novel genus and species of *Diaporthostomataceae* (*Diaporthales*) in China

**DOI:** 10.3897/imafungus.16.145422

**Published:** 2025-03-07

**Authors:** Ning Jiang, Han Xue, Yong Li

**Affiliations:** 1 Key Laboratory of Biodiversity Conservation of National Forestry and Grassland Administration, Ecology and Nature Conservation Institute, Chinese Academy of Forestry, Beijing 100091, China Ecology and Nature Conservation Institute, Chinese Academy of Forestry Beijing China

**Keywords:** *Ascomycota*, biodiversity, phylogeny, systematics, taxonomy

## Abstract

*Diaporthales* is a significant fungal order comprising species that predominantly inhabit plant tissues, being pathogens, endophytes, and saprobes. Recent studies have uncovered extensive species diversity across various hosts, utilizing both morphological characteristics and molecular phylogenetic analyses. In this study, samples of leaf spots and branch cankers were collected from China, and fungal isolations were established. Species identification was conducted using a phylogenetic approach based on combined sequence data from the internal transcribed spacer (ITS) region, large subunit ribosomal DNA (LSU), the DNA-directed RNA polymerase II second largest subunit (*rpb2*), and translation elongation factor 1-alpha (*tef1*) genes, together with morphological observations. As a result, the novel genus *Tiania* is proposed, with three newly described species: *T.chinensis*, *T.lithocarpicola*, and *T.quercicola*. These species are validated by pairwise homoplasy index (PHI) analysis, ensuring robust support for their distinction. This study explores the rare family *Diaporthostomataceae*, providing the first descriptions of their anamorphic forms. By offering detailed morphological and molecular data, this research lays a foundation for future taxonomic and systematic studies of the *Diaporthales*.

## ﻿Introduction

The order *Diaporthales* (*Sordariomycetes*, *Ascomycota*) comprises a diverse and ecologically significant group of fungi, including pathogens, endophytes, and saprotrophs, primarily associated with plant tissues, especially woody hosts (Sogonov et al. 2008; Walker et al. 2010; Voglmayr et al. 2012; Rossman et al. 2015; Zhang et al. 2023). Members of this order are distinguished by their unique teleomorphic features, including solitary or aggregated, immersed or erumpent, orange, brown, or black perithecial ascomata located in stromatic tissues or on substrates, often with a defined centrum; unitunicate asci with a prominent refractive ring; short to elongate, aseptate or septate, hyaline or pigmented ascospores (Castlebury et al. 2002; Rossman et al. 2007; Senanayake et al. 2017; Jaklitsch and Voglmayr 2020). The anamorphic states of this order are highly diverse, encompassing acervular, pycnidial, and synnematal conidiomata; usually phialidic and rarely annellidic conidiogenous cells; unicellular to septate, hyaline to pigmented, various shaped conidia (Castlebury et al. 2002; Senanayake et al. 2017; Fan et al. 2018; Jiang et al. 2021b).

In the traditional morphological classification system, families and genera within *Diaporthales* are primarily distinguished based on the morphology of stromata, including stromatic development and tissue types, the position of ascomata and perithecial necks, and the shape of ascospores. However, taxonomists have held differing views on the classification of diaporthalean fungi into families, e.g., *Diaporthaceae* and *Melanosporaceae* in Luttrell (1951), *Diaporthaceae*, *Gnomoniaceae* and *Melanconidaceae* in Chadefaud (1960), *Diaporthaceae*, *Gnomoniaceae* and *Valsaceae* in Wehmeyer (1975), *Coryneaceae*, *Cytosporaceae*, *Gnomoniaceae* and *Melanconidaceae* in Barr (1978).

Castlebury et al. (2002) initiated the molecular phylogenetic analysis of the order *Diaporthales*, focusing on the evaluation of its familial relationships. Their study included four families, *Diaporthaceae*, *Gnomoniaceae*, *Melanconidaceae*, and *Valsaceae*, along with two complexes: the *Schizoparme* complex and the *Cryphonectria*-*Endothia* complex, based on large subunit nuclear ribosomal DNA (LSU) sequences. Subsequently, additional families, including *Cryphonectriaceae*, *Juglanconidaceae*, *Harknessiaceae*, *Lamproconiaceae*, *Macrohilaceae*, *Pseudoplagiostomaceae*, *Schizoparmaceae*, *Stilbosporaceae*, and *Sydowiellaceae*, were incorporated into *Diaporthales* supported by morphological studies and phylogenetic analyses using the internal transcribed spacer (ITS) and LSU sequences (Gryzenhout et al. 2006; Rossman et al. 2007; Cheewangkoon et al. 2010; Crous et al. 2012b, 2015a; Voglmayr and Jaklitsch 2014; Alvarez et al. 2016; Norphanphoun et al. 2016; Voglmayr et al. 2017; Yang et al. 2017). Senanayake et al. (2017) further advanced the classification of *Diaporthales* by investigating its phylogenetic relationships using a combined dataset of ITS, LSU, DNA-directed RNA polymerase II second largest subunit (*rpb2*), and translation elongation factor 1-alpha (*tef1*) gene regions. Their work expanded the recognized families within this order to 21. Subsequently, additional families were incorporated into *Diaporthales* based on molecular phylogenetic frameworks based on ITS, LSU, *rpb2*, and *tef1* sequence data, as well as morphological characteristics (Senanayake et al. 2018; Fan et al. 2018; Guterres et al. 2019; Jiang et al. 2020, 2021a).

The family *Diaporthostomataceae* was initially proposed by Fan et al. (2018) to include a single genus and species. When first described, it was considered phylogenetically sister to *Diaporthosporellaceae* (Fan et al. 2018). However, Jiang et al. (2020) sequenced the *tef1* gene of *Diaporthosporellacercidicola* and determined that these two families are not sister clades, a finding later corroborated by Mu et al. (2024). *Diaporthostomataceae* is characterized by a typical diaporthalean teleomorph and can be distinguished from other families in *Diaporthales* by the absence of stromatic tissues and their fusoid, multiguttulate ascospores with an inconspicuous median septum (Fan et al. 2018).

Species of *Diaporthales* are well-known for causing plant diseases (Shuttleworth and Guest 2017; Jiang et al. 2019a; Udayanga et al. 2021; Zhu et al. 2024). For example, *Cryphonectriaparasitica* is the causal agent of chestnut blight (Jiang et al. 2019b), while *Gnomoniopsisfragariae*, *Paragnomoniafragariae*, and *Paraphomopsisobscurans* are responsible for strawberry leaf spot (Udayanga et al. 2021). Additionally, many species of *Cytospora* are associated with tree canker diseases (Lawrence et al. 2018). Recent studies combining morphological and molecular phylogenetic analyses have uncovered numerous cryptic species within *Diaporthales* associated with tree disease symptoms (Liu et al. 2024; Mu et al. 2024).

During the extensive investigations conducted to collect forest pathogens in China, several diaporthalean taxa exhibiting branch canker and leaf spot symptoms were successfully isolated. The primary objective of this study was to accurately identify these newly collected diaporthalean species through morphological and molecular methods, while also elucidating their phylogenetic relationships within *Diaporthales*.

## ﻿Materials and methods

### ﻿Surveys and isolations

Samples, including leaf spots and branch cankers, were collected between 2019 and 2024 in China. Leaf samples were placed in self-sealing bags and transported to the laboratory for fungal isolation. Branch samples with visible fruiting bodies were cut into approximately 15 cm segments and preserved in paper sample bags for fungal isolation in the laboratory.

Leaves exhibiting spots were washed under tap water for 20 s, then dried on sterilized absorbent cotton. The leaves were surface sterilized by immersing them for 1 min in 75% ethanol, followed by 3 min in 1.25% sodium hypochlorite, and then for 1 min in 75% ethanol. After a 2-min rinse in distilled water, they were dried again on sterilized absorbent cotton. The leaves were then cut into 0.5 × 0.5 cm pieces using a sterile double-edged blade. Pieces with diseased and healthy tissues were transferred onto the surface of potato dextrose agar (PDA; 200 g potatoes, 20 g dextrose, and 20 g agar per liter) and incubated at 25 °C to obtain pure fungal cultures. Branches with fresh fruiting bodies were rinsed in tap water for 30 s to remove surface dust and then dried on sterilized absorbent cotton. Conidiomata and perithecia were carefully sectioned with a sterile blade to expose the spore masses, which were then transferred onto the surface of PDA plates using a sterile needle. The plates were incubated at 25 °C to establish fungal cultures. Type specimens were deposited in the herbarium of the Chinese Academy of Forestry (CAF, http://museum.caf.ac.cn/), and isolates were stored at the China Forestry Culture Collection Center (CFCC, https://cfcc.caf.ac.cn/).

### ﻿Morphological analyses

The morphology of the new species identified in this study was analyzed based on fruiting bodies naturally formed on branches and PDA plates. Pseudostromata and conidiomata were sectioned using a double-edged blade, and their structures were examined under a Zeiss Discovery V8 stereomicroscope (Jena, Germany). Microscopic features, including asci, ascospores, conidiophores, conidiogenous cells, and conidia, were observed and photographed with an Olympus BX51 microscope (Tokyo, Japan). For spore measurements, 50 spores were randomly selected. The results are presented as maximum and minimum values (in parentheses), along with the range expressed as the mean ± standard deviation.

Colony characteristics were observed on three media types: potato dextrose agar (PDA), malt extract agar (MEA; 30 g malt extract, 5 g mycological peptone, 15 g agar per liter), and synthetic nutrient-deficient agar (SNA; 0.2 g glucose, 0.2 g sucrose, 1 g potassium dihydrogen phosphate, 1 g potassium nitrate, 0.25 g magnesium sulfate anhydrous, 0.5 g potassium chloride, 14 g agar per liter). Colony colors were documented following Rayner (1970).

### ﻿Molecular analyses

Genomic DNA was extracted from colonies grown on PDA plates for 10 d using the Wizard® Genomic DNA Purification Kit (Promega, Madison, WI, USA), following the manufacturer’s protocol. To amplify the ITS, LSU, *rpb2* and *tef1* gene loci, the following primer pairs were used: ITS1/ITS4, LR0R/LR5, RPB2-5F/fRPB2-7cR, and EF1-728F/EF1-986R or EF1-728F/EF2, respectively (White et al. 1990; Glass and Donaldson 1995; Carbone and Kohn 1999; Liu et al. 1999; Rehner et al. 2001). Polymerase chain reaction (PCR) conditions included an initial denaturation at 94 °C for 5 min, followed by 35 cycles of 30 s at 94 °C, 50 s at 48 °C (for ITS and LSU) or 54 °C (for *tef1*) or 55 °C (for *rpb2*), and 1 min at 72 °C, with a final elongation step of 7 min at 72 °C. Amplicons were sequenced in both directions by Ruibo Xingke Biotechnology Company Limited (Beijing, China).

Sequences were assembled using Seqman v. 7.1.0 (DNASTAR Inc., Madison, WI, USA) and deposited in GenBank, and reference sequences were selected from recent studies on *Diaporthales* (Table 1). Sequence alignments of the four loci (ITS, LSU, *rpb2*, and *tef1*) were performed in MAFFT v. 7 (Katoh and Standley 2023) and manually edited in MEGA v. 7.0.21.

**Table 1.** Details of isolates included in the molecular study.

Species	Strain	GenBank accession numbers				References
		ITS	LSU	*rpb2*	*tef1*	
*Apiognomoniaerrabunda*	AR 2813	DQ313525	NA	DQ862014	DQ313565	Sogonov et al. 2007
*Apiosporopsiscarpinea*	CBS 771.79	NA	AF277130	NA	NA	Zhang and Blackwell 2001
*Apoharknessiainsueta*	CBS 111377*	JQ706083	AY720814	NA	MN271820	Crous et al. 2012b; Jiang et al. 2020
*Apoharknessiainsueta*	CBS 114575	MN172402	MN172370	NA	MN271821	Jiang et al. 2020
*Asterosporiumasterospermum*	MFLU 15-3555	NA	MF190062	NA	NA	Senanayake et al. 2017
*Auratiopycnidiellatristaniopsis*	CBS 132180*	JQ685516	JQ685522	NA	MN271825	Crous et al. 2012a; Jiang et al. 2020
*Auratiopycnidiellatristaniopsis*	CPC 16371	MN172405	MN172374	NA	MN271826	Jiang et al. 2020
*Aurifilummarmelostoma*	CBS 124928*	FJ890495	MH874934	MN271788	MN271827	Begoude et al. 2010; Jiang et al. 2020
*Chrysofoliabarringtoniae*	TBRC 5647*	KU948046	KU948045	NA	NA	Suwannarach et al. 2016
*Chrysofoliacolombiana*	CBS 139909*	KR476738	KR476771	NA	MN271829	Crous et al. 2015b; Jiang et al. 2020
*Coniellaafricana*	CBS 114133*	AY339344	AY339293	KX833421	KX833600	Alvarez et al. 2016
*Coniellaeucalyptorum*	CBS 112640*	AY339338	AY339290	KX833452	KX833637	Alvarez et al. 2016
*Coniellafusiformis*	CBS 141596*	KX833576	KX833397	KX833481	KX833674	Alvarez et al. 2016
*Coryneumgigasporum*	CFCC 52319*	MH683565	MH683557	MH685729	MH685737	Jiang et al. 2018b
*Coryneumumbonatum*	D201	MH674329	MH674329	MH674333	MH674337	Jiang et al. 2018b
*Cryphonectriacitrine*	CBS 109758*	MN172407	EU255074	EU219342	MN271843	Sogonov et al. 2008; Jiang et al. 2020
*Cryphonectriadecipens*	CBS 129351	EU442657	MN172385	MN271796	MN271844	Jiang et al. 2020
*Cytosporachrysosperma*	CFCC 89982	KP281261	KP310805	KU710952	KP310848	Yang et al. 2015
*Cytosporaelaeagni*	CFCC 89633	KF765677	KF765693	KU710956	KU710919	Yang et al. 2015
*Cytosporaviridistroma*	CBS 202.36*	MN172408	MN172388	NA	MN271853	Jiang et al. 2020
*Dendrostomacastaneae*	CFCC 52745*	MH542488	MH542644	MH545395	MH545437	Jiang et al. 2019a
*Dendrostomachinense*	CFCC 52755*	MH542500	MH542648	MH545407	MH545449	Jiang et al. 2019a
*Diaportheeres*	LC3198	KP267873	KY011845	NA	KP267947	Gao et al. 2016
*Diaporthehongkongensis*	LC0784	KC153104	KY011876	NA	KC153095	Gao et al. 2016
*Diaporthosporellacercidicola*	CFCC 51994*	KY852492	KY852515	NA	MN271855	Yang et al. 2017; Jiang et al. 2020
*Diaporthosporellamacarangae*	NCYU 19-0359*	MW114354	MW114415	NA	NA	Tennakoon et al. 2021
*Diaporthosporellamacarangae*	NCYU 19-0363	MW114355	MW114416	NA	NA	Tennakoon et al. 2021
*Diaporthostomamachili*	CFCC 52100*	MG682080	MG682020	MG682040	MG682060	Fan et al. 2018
*Diaporthostomamachili*	CFCC 52101	MG682081	MG682021	MG682041	MG682061	Fan et al. 2018
*Disculoideseucalyptorum*	CBS 132184	JQ685518	JQ685524	MH545414	MH545456	Crous et al. 2012a
*Dwiroopalythri*	CBS 109755*	MN172410	MN172389	MN271801	MN271859	Jiang et al. 2020
*Dwiroopapunicae*	CBS 143163*	MK510676	MK510686	MK510692	NA	Xavier et al. 2019
*Foliocryphiaeucalypti*	CBS 124779*	GQ303276	GQ303307	MN271802	MN271861	Cheewangkoon et al. 2009; Jiang et al. 2020
*Foliocryphiaeucalyptorum*	CBS 142536*	KY979772	KY979827	MN271803	MN271862	Crous et al. 2013; Jiang et al. 2020
*Gnomoniagnomon*	CBS 199.53	DQ491518	AF408361	EU219295	NA	Castlebury et al. 2002; Sogonov et al. 2007
*Harknessiaaustraliensis*	CBS 132119*	JQ706085	JQ706211	NA	MN271863	Crous et al. 2012b; Jiang et al. 2020
*Harknessiacapensis*	CBS 111829*	AY720719	AY720816	NA	MN271864	Crous et al. 2012b; Jiang et al. 2020
*Harknessiagibbosa*	CBS 120033*	EF110615	EF110615	NA	MN271868	Crous et al. 2012b; Jiang et al. 2020
*Juglanconisjuglandina*	CBS 121083	KY427148	KY427148	KY427198	KY427217	Voglmayr et al. 2017
*Juglanconisoblonga*	MAFF 410216	KY427153	KY427153	KY427203	KY427222	Voglmayr et al. 2017
*Juglanconispterocaryae*	MAFF 410079	KY427155	KY427155	KY427205	KY427224	Voglmayr et al. 2017
*Lamproconiumdesmazieri*	MFLUCC 15-0870	KX430134	KX430135	MF377605	MF377591	Norphanphoun et al. 2016; Senanayake et al. 2017
*Lamproconiumdesmazieri*	MFLUCC 15-0872	KX430138	KX430139	MF377606	MF377593	Norphanphoun et al. 2016; Senanayake et al. 2017
*Luteocirrhusshearii*	CBS 130776*	KC197021	KC197019	MN271807	MN271890	Crane and Burgess 2013; Jiang et al. 2020
*Macrohilumeucalypti*	CPC 10945	DQ195781	DQ195793	MN271809	NA	Crous et al. 2006; Jiang et al. 2020
*Macrohilumeucalypti*	CPC 19421	KR873244	KR873275	MN271810	NA	Crous et al. 2006; Jiang et al. 2020
*Mastigosporellaanisophylleae*	CBS 136421*	KF779492	KF777221	NA	MN271892	Crous et al. 2013; Jiang et al. 2020
*Mastigosporellapigmentata*	COAD 2370*	MG587929	MG587928	NA	NA	Crous et al. 2018
*Melanconiellaellisii*	BPI 878343	JQ926271	JQ926271	JQ926339	JQ926406	Voglmayr et al. 2012
*Melanconiellaspodiaea*	MSH	JQ926298	JQ926298	JQ926364	JQ926431	Voglmayr et al. 2012
*Melanconisbetulae*	CFCC 50471	KT732952	KT732971	KT732984	KT733001	Fan et al. 2016
*Melanconisitoana*	CFCC 50474	KT732955	KT732974	KT732987	KT733004	Fan et al. 2016
*Melanconisstilbostoma*	CFCC 50475	KT732956	KT732975	KT732988	KT733005	Fan et al. 2016
*Nakataeaoryzae*	CBS 243.76	KM484861	DQ341498	NA	NA	Klaubauf et al. 2014
*Neocryphonectriachinensis*	CFCC 53025*	MN172414	MN172397	MN271812	MN271893	Jiang et al. 2020
*Neopseudomelanconiscastaneae*	CFCC 52787*	MH469162	MH469164	NA	NA	Jiang et al. 2018a
*Phaeoappendicosporathailandensis*	MFLU 12-2131	MF190157	MF190102	NA	NA	Senanayake et al. 2017
*Prosopidicolaalbizziae*	CPC 27478	KX228274	KX228325	NA	NA	Crous et al. 2013
*ProsopidicolaMexicana*	CBS 113529	AY720709	NA	NA	NA	Lennox et al. 2004
*Pseudomelanconiscaryae*	CFCC 52110*	MG682082	MG682022	MG682042	MG682062	Fan et al. 2018
*Pseudoplagiostomacorymbiae*	CPC 14161	GU973510	GU973604	NA	GU973540	Cheewangkoon et al. 2010
*Pseudoplagiostomaoldie*	CBS 115722	GU973535	GU973610	NA	GU973565	Cheewangkoon et al. 2010
*Pseudoplagiostomavariabile*	CBS 113067	GU973536	GU973611	NA	GU973566	Cheewangkoon et al. 2010
*Pyriculariagrisea*	Ina168	NA	AB026819	NA	NA	Sone et al. 2000
*Pyrisporacastaneae*	CFCC 54349*	MW208108	MW208105	MW218535	MW227340	Jiang et al. 2021a
*Pyrisporacastaneae*	CFCC 54350	MW208109	MW208106	MW218536	MW227341	Jiang et al. 2021a
*Silliakarstenii*	MFLU 16-2864	KY523482	KY523500	KY501636	NA	Senanayake et al. 2017
*Sirococcustsugae*	CBS 119626	EU199203	EU199136	EU199159	EF512534	Mejía et al. 2008
*Stegonsporiumacerophilum*	CBS 117025	EU039982	EU039993	KF570173	EU040027	Voglmayr and Jaklitsch 2008, 2014
*Stilbosporalongicornuta*	CBS 122529*	KF570164	KF570164	KF570194	KF570232	Voglmayr and Jaklitsch 2014
*Synnemasporellaaculeans*	CFCC 52094	MG682086	MG682026	MG682046	MG682066	Fan et al. 2018
*Synnemasporellatoxicodendri*	CFCC 52097*	MG682089	MG682029	MG682049	MG682069	Fan et al. 2018
***Tianiachinensis***	CFCC 59134*	**PQ781258**	**PQ781255**	**PQ786769**	**PQ786772**	In this study
***Tianiachinensis***	**CFCC 59135**	**PQ781259**	**PQ781256**	**PQ786770**	**PQ786773**	In this study
***Tianiachinensis***	CFCC 71190*	**PQ781260**	**PQ781257**	**PQ786771**	**PQ786774**	In this study
***Tianialithocarpicola***	CFCC 55331*	**OK339758**	**OK339787**	**OK358595**	**OK358599**	In this study
***Tianialithocarpicola***	**CFCC 55882**	**OK339759**	**OK339788**	**OK358596**	**OK358600**	In this study
***Tianiaquercicola***	CFCC 54435*	**OK339756**	**OK339785**	**OK358593**	**OK358597**	In this study
***Tianiaquercicola***	**CFCC 55885**	**OK339757**	**OK339786**	**OK358594**	**OK358598**	In this study
*Tubakiaiowensis*	CBS 129012*	MG591879	MG591971	NA	MG592064	Braun et al. 2018
*Tubakiaseoraksanensis*	CBS 127490*	MG591907	KP260499	NA	MG592094	Braun et al. 2018

Note. Strains from this study are in bold, NA means not available and * represents the ex-type strains.

Phylogenetic analyses were conducted on the combined dataset of the four loci using Maximum Likelihood (ML) and Bayesian Inference (BI). ML analysis was performed with the GTR substitution model and 1000 bootstrap replicates via the CIPRES Science Gateway portal (https://www.phylo.org/; Miller et al. 2010) using RAxML-HPC BlackBox v. 8.2.10 (Stamatakis 2008). BI analysis applied partition-specific evolutionary models selected by MrModeltest v. 2.3 using the Akaike Information Criterion (AIC). Markov Chain Monte Carlo (MCMC) simulations in MrBayes v. 3.1.2 (Ronquist and Huelsenbeck 2003) were run for 10 million generations with two chains initiated from random trees. Convergence was confirmed by an average standard deviation of split frequencies below 0.01, and trees were sampled every 1000 generations. The first 25% of sampled trees were discarded as burn-in, and posterior probabilities (BPP) were calculated from the remaining trees. Bootstrap (BS) support in ML analyses was assessed with 1000 replicates, and phylogenetic trees were visualized in FigTree v. 1.4.4 (Rambaut 2018).

The pairwise homoplasy index (PHI, Φ_w_) test was conducted using the SplitsTree App to evaluate recombination among closely related phylogenetic species (Huson and Bryant 2024). The analysis used a concatenated four-locus dataset (ITS, LSU, *rpb2*, and *tef1*), and Φ_w_-statistic below 0.05 (*p*-value < 0.05) indicated no significant evidence of recombination. Relationships among closely related taxa were illustrated with split graphs generated using the Log-Det transformation and split decomposition methods, providing a clear visualization of phylogenetic associations.

## ﻿Results

### ﻿Phylogenetic analyses

**Figure 1.**
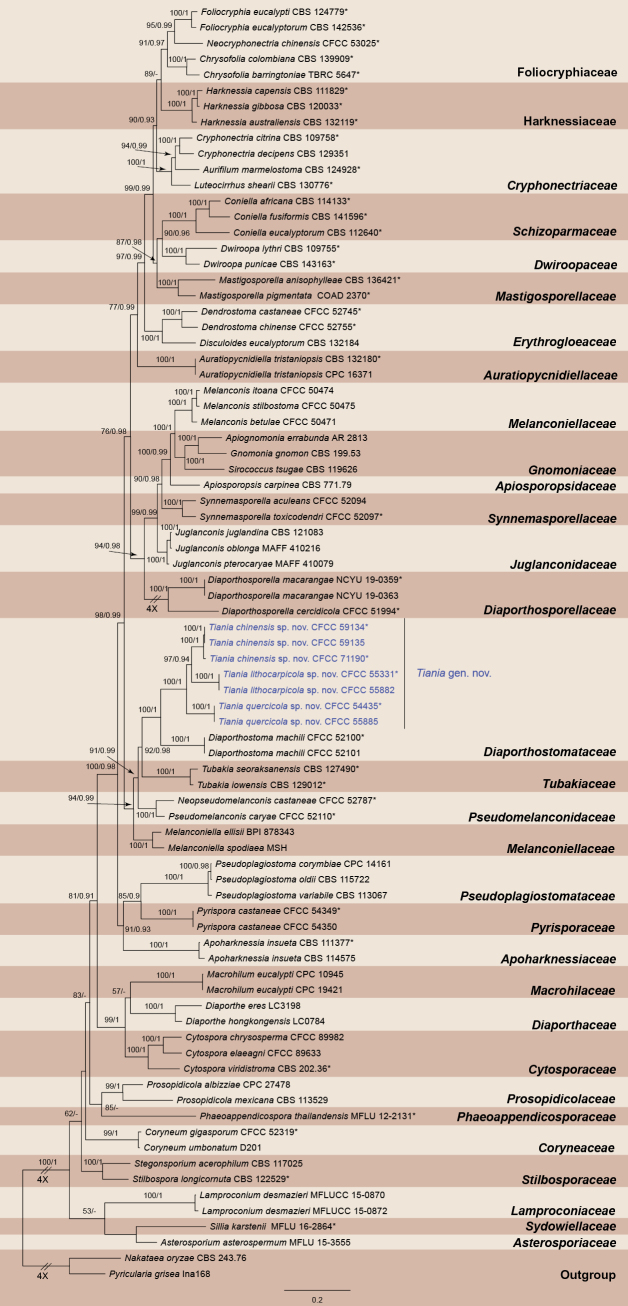
Phylogram of *Diaporthales* resulting from a maximum likelihood analysis based on the ITS, LSU, *rpb2* and *tef1* gene sequence. Numbers above the branches indicate ML bootstrap values (left, MLBS ≥ 50%) and Bayesian posterior probabilities (right, BPP ≥ 0.90). The tree is rooted with *Nakataeaoryzae* (CBS 243.76) and *Pyriculariagrisea* (Ina168). Isolates from the present study are marked in blue.

The combined dataset of ITS, LSU, *rpb2*, and *tef1* comprised 81 strains, with *Nakataeaoryzae* (CBS 243.76) and *Pyriculariagrisea* (Ina168) designated as the outgroup taxa. The final alignment consisted of 3,229 characters (ITS: 660; LSU: 789; *rpb2*: 1,065; *tef1*: 715), including gaps. The ML optimization likelihood value for the best RAxML tree was -53,777.19, with the matrix containing 2,052 distinct alignment patterns and 38.12% undetermined characters or gaps. The estimated base frequencies were A = 0.238237, C = 0.263656, G = 0.272107, and T = 0.226000. Substitution rates were calculated as follows: AC = 1.555398, AG = 2.732713, AT = 1.808604, CG = 1.204566, CT = 6.521498, and GT = 1.000000. The gamma distribution shape parameter (α) was 0.269658. For Bayesian inference (BI) analysis, the most appropriate models for each locus were confirmed using MrModeltest. The selected models were SYM+I+G4 for ITS, SYM+R3 for LSU, TN+F+I+G4 for *rpb2*, and TIM2+F+I+G4 for *tef1*. The Bayesian analysis results aligned with the ML tree topology. ML bootstrap support values (BS) ≥ 50% and Bayesian posterior probabilities (BPP) ≥ 0.90 are indicated on the branches in Fig. 1. The phylogram, constructed using four gene markers, delineated 31 distinct lineages corresponding to 31 families within *Diaporthales*. Phylogenetic analysis revealed that *Diaporthostomataceae* forms a sister clade to *Tubakiaceae*, together constituting a well-supported clade with *Pseudomelanconidaceae* and *Melanconiellaceae*. Notably, the new strains in this study form a robustly supported clade that is sister to *Diaporthostoma*, representing a newly discovered genus which we propose to name *Tiania*.

### ﻿PHI Analysis

**Figure 2.**
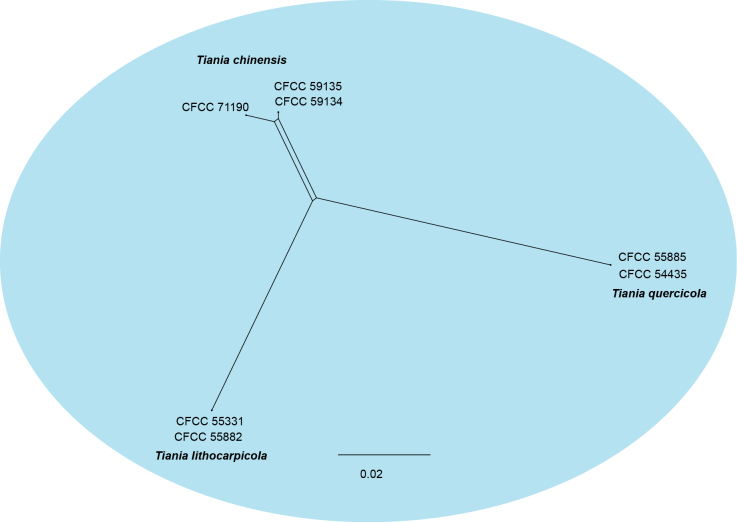
The split graphs of a PHI test result of *Tiania* species using the LogDet transformation and split decomposition options based on the ITS, LSU, *rpb2* and *tef1* gene sequence (*p* = 0.1139).

In the genus *Tiania*, seven isolates were grouped into three distinct clades with high support values (Fig. 1). To validate the species delineation, PHI analysis was performed. The analysis revealed no significant evidence of genetic recombination among these species (*p* = 0.1139; Fig. 2).

### ﻿Taxonomy

#### ﻿ Tiania

Taxon classificationFungiDiaporthalesDiaporthostomataceae

Ning Jiang gen. nov.

16688A0C-EDF9-5EF3-96B8-58C0CA6917EA

857019

##### Etymology.

In honor of Chinese taxonomist Prof. Dr. Chengming Tian for his contributions for forest pathogens.

##### Type species.

*Tianiachinensis* Ning Jiang.

##### Description.

***Pseudostromata*** immersed to semi-immersed in the bark, scattered, conical, with perithecia arranged irregular. ***Ectostromatic disc*** grey to brown, circular to ovoid. ***Ostioles*** brown to black. ***Perithecia*** flask-shaped to spherical. ***Asci*** hyaline, with chitinoid, refractive ring, clavate to elongate-obovoid, 8-spored. ***Ascospores*** biseriate, cylindrical to allantoid, thin-walled, hyaline, 0–1 septate. ***Conidiomata*** acervular in tree branches and sporodochial in culture, aggregated, immersed to semi-immersed, pulvinate. ***Conidiophores*** indistinct, usually reduced to conidiogenous cells. ***Conidiogenous cells*** hyaline, smooth, cylindrical to ampulliform, phialidic. ***Conidia*** aseptate, hyaline, smooth, multi-guttulate, fusoid, cylindrical to allantoid, constricted at the middle or not.

##### Notes.

The newly proposed genus *Tiania* is phylogenetically closely related to *Diaporthostoma* within the family *Diaporthostomaceae* (Fig. 1). Morphologically, *Tiania* can be distinguished from *Diaporthostoma* by its formation of stromatic tissue and its cylindrical to allantoid ascospores (Fan et al. 2018).

#### ﻿ Tiania chinensis

Taxon classificationFungiDiaporthalesDiaporthostomataceae

Ning Jiang sp. nov.

58F314DD-3D18-5640-95C7-488DAD65261C

857020

Figs 3 , 4

##### Etymology.

Named after the collection country of the type specimen, China.

##### Diagnosis.

Distinct from its phylogenetically related species *T.lithocarpicola* by longer conidia.

##### Typus.

CHINA • Xizang Autonomous Region, Rikaze City, Jilong County, Jilong Town, Rema Village, on diseased branches of *Quercussemecarpifolia*, 20 August 2022, Ning Jiang, Min Liu & Peng Jin (**holotype** CAF800088; ex-holotype culture CFCC 59134); • Xizang Autonomous Region, Linzhi City, Gongbujiangda County, Gongbujiangda Town, on diseased branches of *Quercusspinosa*, 7 July 2024, Ning Jiang, Jiangrong Li, Jieting Li & Liangna Guo (**paratype** CAF800141; ex-paratype culture CFCC 71190).

**Figure 3.**
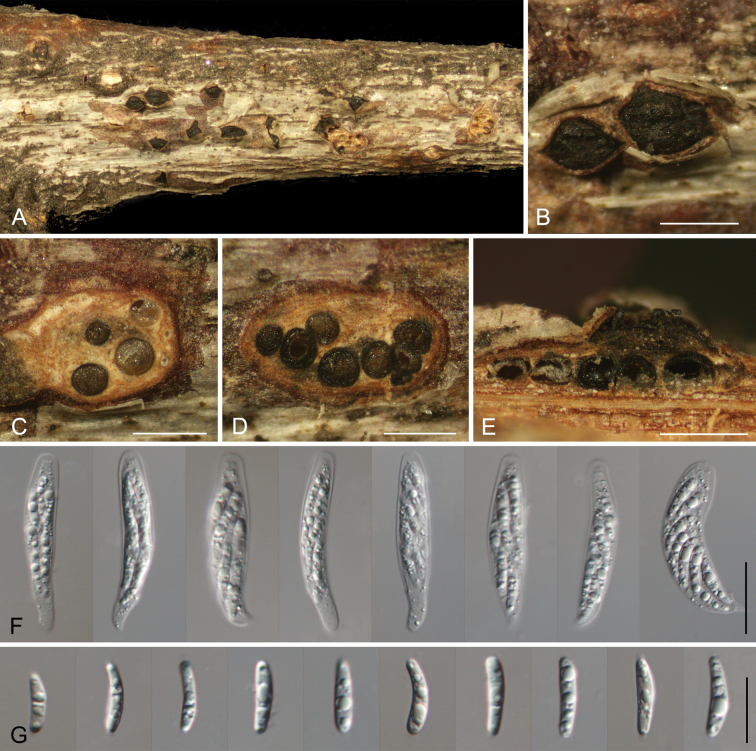
*Tianiachinensis* sp. nov. from *Quercusspinosa* (CAF800141). **A, B** Habit of ascostromata on branch; **C, D** transverse section through ascostroma; **E** longitudinal section through ascostroma; **F** asci; **G** ascospores. Scale bars: 500 μm (**B–E**); 20 μm (**F**); 10 μm (**G**).

##### Description.

***Pseudostromata*** immersed to semi-immersed in the bark, scattered, conical, 630–1240 μm diam, 330–480 μm high, with 5–10 perithecia arranged irregularly. ***Ectostromatic disc*** grey to brown, circular to ovoid, 300–470 μm diam. ***Ostioles*** brown to black, 90–150 μm diam. ***Perithecia*** flask-shaped to spherical, 490–620 μm diam. ***Asci*** hyaline, with chitinoid, refractive ring, clavate to elongate-obovoid, (38.5–)41.5–47.5(–52) × (7–)8–9.5(–10) μm, 8-spored. ***Ascospores*** biseriate, cylindrical to allantoid, thin-walled, hyaline, 0–1 septate, (11–)11.5–14.5(–15) × (2.5–)3–3.5 (n = 50) μm, L/W ratio = 3.7–5. ***Conidiomata*** acervular, aggregated, immersed to semi-immersed in the bark, pulvinate, dark brown to black, 250–600 μm high, 350–1000 μm diam. ***Conidiophores*** indistinct, usually reduced to conidiogenous cells. ***Conidiogenous cells*** hyaline, smooth, cylindrical, phialidic, 9.2–14.5 × 2–2.9 μm. ***Conidia*** aseptate, hyaline, smooth, multi-guttulate, cylindrical to allantoid, straight or slightly curved, (7.5–)8–9(–10) × (2–)2.5–3(–3.5) μm (n = 50), L/W = 2.7–3.6.

**Figure 4.**
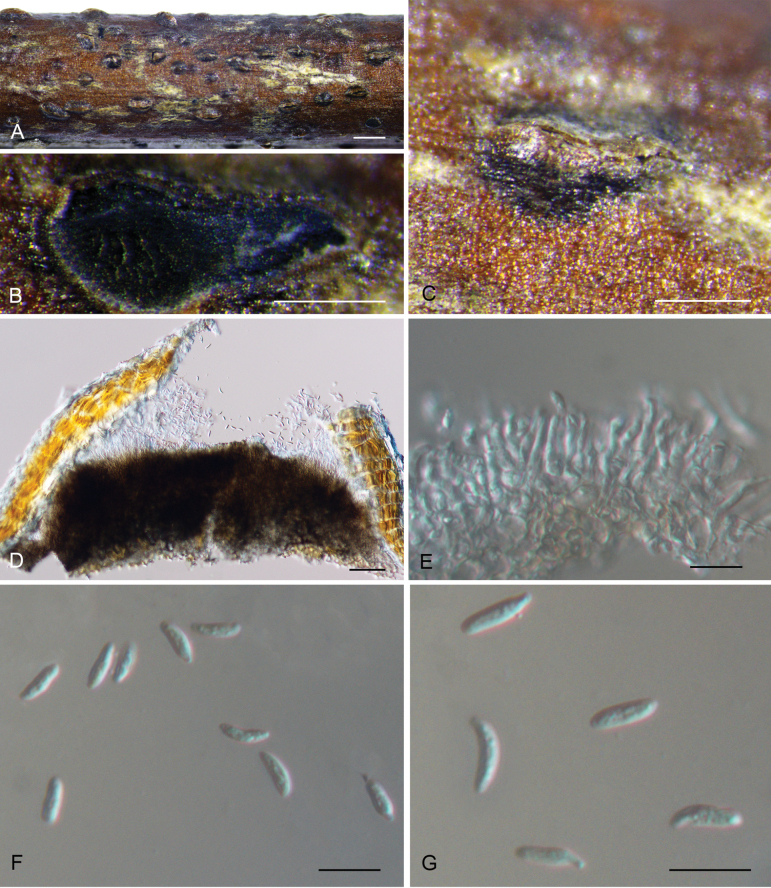
*Tianiachinensis* sp. nov. from *Quercussemecarpifolia* (CAF800088). **A, C** Habit of conidiomata on branch; **B** transverse section through conidioma; **D** longitudinal section through conidioma; **E** conidiogenous cells with attached conidia; **F, G** conidia. Scale bars: 500 μm (**A**); 200 μm (**B, C**); 100 μm (**D**); 10 μm (**E–G**).

##### Culture characteristics.

***Colonies on PDA*** flat, spreading, with abundant flocculent aerial mycelium and even margin, white to sky grey, reaching 90 mm diam after 2 wk at 25 °C. ***Colonies on MEA*** flat, spreading, lavender grey to grey olivaceous, reaching 90 mm diam after 2 wk at 25 °C. ***Colonies on SNA*** flat, spreading, with sparse flocculent aerial mycelium and feathery margin.

##### Additional material examined.

CHINA • Xizang Autonomous Region, Rikaze City, Jilong County, Jilong Town, Rema Village, from cankered barks of *Quercussemecarpifolia*, 21 August 2022, Ning Jiang, Min Liu & Peng Jin (living culture CFCC 59135).

##### Distribution.

China, Xizang Autonomous Region.

##### Ecology.

Associated with branch canker disease with *Quercussemecarpifolia* and *Q.spinosa*.

##### Notes.

Three isolates obtained from diseased branches of *Quercussemecarpifolia* and *Q.spinosa* formed a distinct clade, separate from *Tianialithocarpicola* and *T.quercicola*, and are identified as *T.chinensis* sp. nov. This species can be distinguished from *T.lithocarpicola* by its longer conidia (8–9 × 2.5–3 μm in *T.chinensis* vs. 5–6.5 × 2–2.5 μm in *T.lithocarpicola*) and from *T.quercicola* by its cylindrical to allantoid conidia.

#### ﻿ Tiania lithocarpicola

Taxon classificationFungiDiaporthalesDiaporthostomataceae

Ning Jiang sp. nov.

1AC1AF3F-38A0-5D7C-A855-BB841F40FF12

857021

Fig. 5

##### Etymology.

Named after the host genus *Lithocarpus* and “-*cola*” = “inhabiting”.

##### Diagnosis.

Distinct from its sister species *T.chinensis* by shorter conidia.

##### Typus.

CHINA • Hainan Province, Changjiang Li Autonomous County, Bawangling National Forest Park, on diseased leaves of *Lithocarpuselaeagnifolius*, 12 November 2018, Yong Li (**holotype** CAF800042; ex-holotype culture CFCC 55331).

##### Description.

***Conidiomata*** in culture sporodochial, aggregated, erumpent, pulvinate, light brown, 150–650 μm diam., exuding light brown conidial masses. ***Conidiophores*** hyaline, smooth, cylindrical, branched. ***Conidiogenous cells*** hyaline, smooth, cylindrical to ampulliform, attenuate towards the apex, phialidic, 6.5–22.5 × 1.5–2.5 μm. ***Conidia*** aseptate, hyaline, smooth, multi-guttulate, fusoid to ellipsoid, straight or slightly curved, (4.5–)5–6.5(–7.5) × 2–2.5(–3) μm (n = 50), L/W = 1.9–3.5.

**Figure 5.**
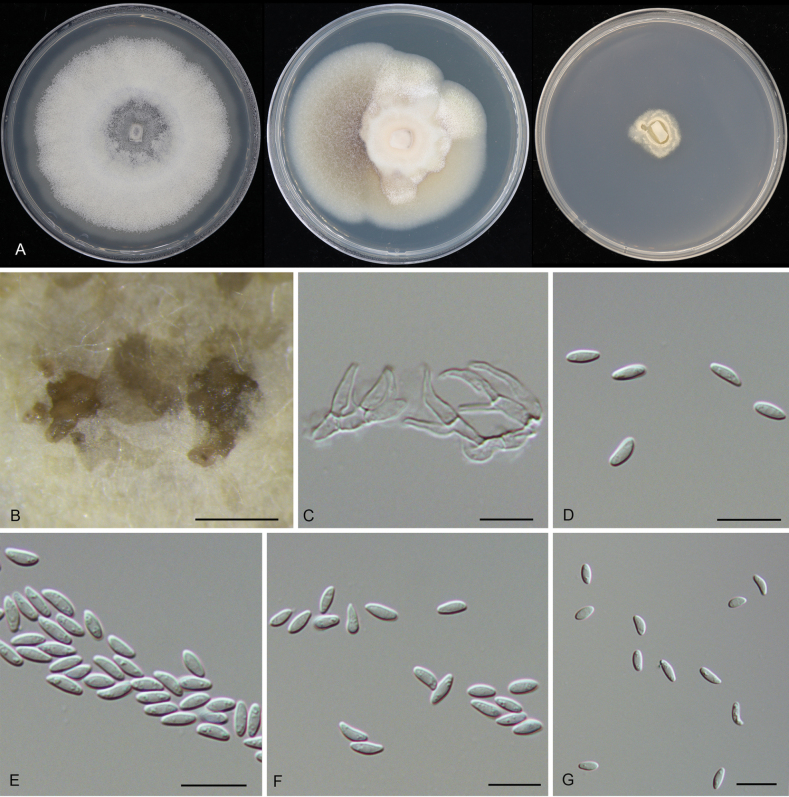
*Tianialithocarpicola* sp. nov. from *Lithocarpuselaeagnifolius* (CAF800042). **A** Colonies on PDA, MEA and SNA after 10 d at 25 °C; **B** conidiomata formed on PDA; **C** conidiogenous cells; **D–G** conidia. Scale bars: 300 μm (**B**); 10 μm (**C–G**).

##### Culture characteristics.

***Colonies on PDA*** flat, spreading, with moderate flocculent aerial mycelium and even margin, forming concentric rings, white to straw, reaching 90 mm diam after 2 wk at 25 °C. ***Colonies on MEA*** flat, spreading, with moderate flocculent aerial mycelium and undulating margin, forming salmon irregular center area and smoke grey to ochreous outer area, reaching 70 mm diam after 2 wk at 25 °C. ***Colonies on SNA*** flat, dense, white, slowly growing.

##### Additional material examined.

CHINA • Hainan Province, Changjiang Li Autonomous County, Bawangling National Forest Park, from leaf spots of *Lithocarpuselaeagnifolius*, 12 November 2018, Yong Li (living culture CFCC 55882).

##### Distribution.

China, Hainan Province.

##### Ecology.

Associated with leaf spot disease with *Lithocarpuselaeagnifolius*.

##### Notes.

*Tianialithocarpicola* is phylogenetically closely related to *T.chinensis* but can be distinguished by its shorter conidia (5–6.5 × 2–2.5 μm in *T.lithocarpicola* vs. 8–9 × 2.5–3 μm in *T.chinensis*).

#### ﻿ Tiania quercicola

Taxon classificationFungiDiaporthalesDiaporthostomataceae

Ning Jiang sp. nov.

CF16E0E8-C425-51D8-9594-1221B9C4C4DF

857020

Fig. 6

##### Etymology.

Named after the host genus *Quercus* and “-*cola*” = “inhabiting”.

##### Diagnosis.

Distinct from *T.chinensis* and *T.lithocarpicola* by conidia that are constricted at the middle.

##### Typus.

CHINA • Hainan Province, Changjiang Li Autonomous County, Bawangling National Forest Park, on diseased leaves of *Quercusmacrocalyx*, 30 March 2019, Yong Li (**holotype** CAF800035; ex-holotype culture CFCC 54435).

##### Description.

***Conidiomata*** in culture sporodochial, aggregated, erumpent, pulvinate, light orange, 150–700 μm diam., exuding light orange conidial masses. ***Conidiophores*** hyaline, smooth, cylindrical, branched, usually reduced to conidiogenous cells. ***Conidiogenous cells*** hyaline, smooth, cylindrical to ampulliform, attenuate towards the apex, phialidic, 10.5–21.5 × 1–2.5 μm. ***Conidia*** aseptate, hyaline, smooth, multi-guttulate, cylindrical, constricted at the middle, straight or slightly curved, base truncate, 5.5–7(–8) × 2–2.5 μm (n = 50), L/W = 1.6–2.7.

**Figure 6.**
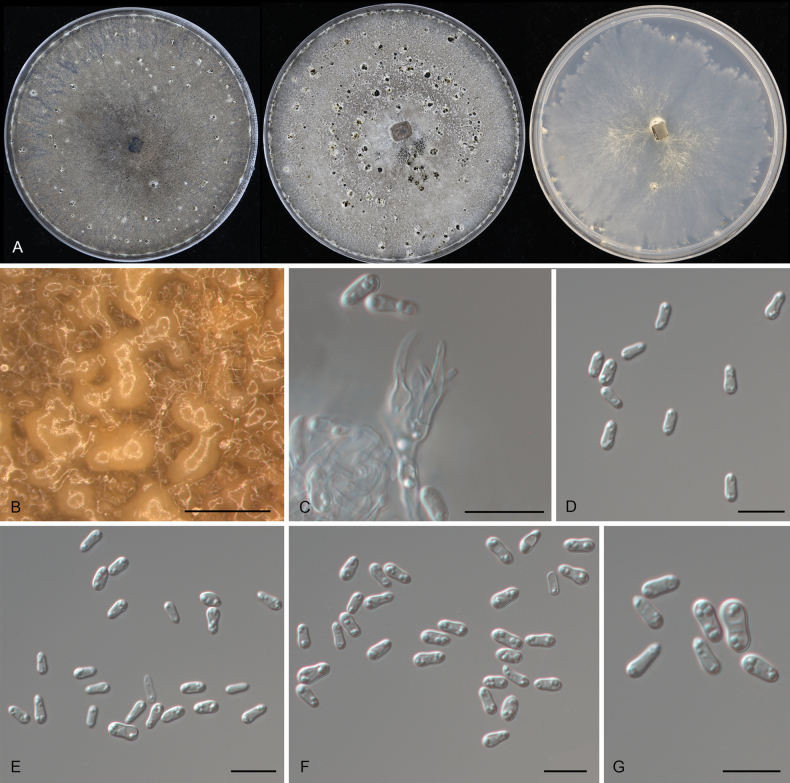
*Tianiaquercicola* sp. nov. from *Quercusmacrocalyx* (CAF800035). **A** Colonies on PDA, MEA and SNA after 10 d at 25 °C; **B** conidiomata formed on PDA; **C** conidiogenous cells; **D–G** conidia. Scale bars: 300 μm (**B**); 10 μm (**C–G**).

##### Culture characteristics.

***Colonies on PDA*** flat, spreading, with abundant flocculent aerial mycelium and even margin, initially white, becoming umber after 1 wk, reaching 90 mm diam after 2 wk at 25 °C. ***Colonies on MEA*** flat, spreading, with abundant flocculent aerial mycelium and undulating margin, white to smoke grey, reaching 90 mm diam after 2 wk at 25 °C. ***Colonies on SNA*** flat, spreading, with sparse flocculent aerial mycelium and feathery margin, white, reaching 90 mm diam after 3 wk at 25 °C.

##### Additional material examined.

CHINA • Hainan Province, Changjiang Li Autonomous County, Bawangling National Forest Park, from leaf spots of *Quercusmacrocalyx*, 30 March 2019, Yong Li (living culture CFCC 55885).

##### Distribution.

China, Hainan Province.

##### Ecology.

Associated with leaf spot disease with *Quercusmacrocalyx*.

##### Notes.

*Tianiaquercicola*, isolated from *Quercusmacrocalyx*, is phylogenetically closely related to *T.chinensis* from *Quercussemecarpifolia* and *Q.spinosa*, and *T.lithocarpicola* from *Lithocarpuselaeagnifolius* (Fig. 1). However, *T.quercicola* can be distinguished from these two species by its conidia, which are constricted at the middle.

### ﻿Key to genera and species of *Diaporthostomataceae*

**Table:** 

1	Stromata well-developed	***Diaporthostomamachili***
–	Stromata absent	**2**
2	Conidia constricted at the middle	***Tianiaquercicola***
–	Conidia not constricted at the middle	**3**
3	Conidia cylindrical to allantoid, 8–9 × 2.5–3 μm	***T.chinensis***
–	Conidia fusoid to ellipsoid, 5–6.5 × 2–2.5 μm	***T.lithocarpicola***

## ﻿Discussion

*Diaporthales* is a well-studied order within *Ascomycota*, both morphologically and phylogenetically (Barr 1978; Voglmayr and Jaklitsch 2014; Alvarez et al. 2016; Senanayake et al. 2017, 2018; Voglmayr et al. 2017). Since the first molecular study on this order, which was based on the single locus LSU (Castlebury 2002), a more robust classification system has been established primarily based on the morphology of naturally formed fruiting bodies on hosts and the phylogeny of combined loci, including ITS, LSU, *rpb2*, and *tef1* (Senanayake et al. 2017; Fan et al. 2018; Jiang et al. 2020). In this study, we introduce a new genus, *Tiania*, in the family *Diaporthostomataceae*, based on newly collected samples exhibiting typical diaporthalean characteristics. Additionally, we propose three new species within this genus, named *T.chinensis*, *T.lithocarpicola*, and *T.quercicola*.

The family *Diaporthostomataceae* was established with a single genus and species, *Diaporthostomamachili* (Fan et al. 2018). It is characterized by a teleomorph resembling *Diaporthe* and can be distinguished from its phylogenetic sister clade, *Diaporthosporellaceae*, by having discrete perithecia and fusoid, straight to curved ascospores with a median septum (Yang et al. 2017; Fan et al. 2018). Unfortunately, no anamorph has been reported for this fungus to date.

As the second genus in the *Diaporthostomataceae* family, *Tiania* is characterized by both teleomorphic and anamorphic states. This genus features pseudostromata resembling those of *Cytospora*, but it is distinguished by its aseptate or septate ascospores, differing from *Cytospora* (Fan et al. 2020). In its anamorphic state, *Tiania* exhibits conidia of various shapes. For instance, *T.chinensis* produces cylindrical to allantoid conidia, similar to those of *Cytospora* (Fan et al. 2020); *T.lithocarpicola* forms fusoid to ellipsoid conidia, resembling those of *Diaporthe* (Dissanayake et al. 2024); and *T.quercicola* develops cylindrical conidia constricted in the middle, akin to those of *Micromelanconis* (Jiang et al. 2021c). Additionally, its dark acervular conidiomata bear a striking resemblance to those of *Coryneum* (Senanayake et al. 2017). Thus, the new genus *Tiania* exhibits morphological similarities to several taxa, encompassing features from different families within *Diaporthales*.

The taxonomy of *Diaporthales* has undergone significant advancements over the past decade, uncovering numerous fascinating taxa (Voglmayr and Jaklitsch 2014; Alvarez et al. 2016; Senanayake et al. 2017, 2018; Voglmayr et al. 2017; Fan et al. 2018; Jiang et al. 2020, 2021b). At the family level, several families, including *Cytosporaceae*, *Diaporthaceae*, and *Gnomoniaceae*, are large groups containing a high number of species (Senanayake et al. 2018). In contrast, some families, such as *Dwiroopaceae*, *Mastigosporellaceae*, and *Diaporthosporellaceae*, are relatively less studied and comprise only a small number of species (Fan et al. 2018; Xavier et al. 2019; Jiang et al. 2020). The family *Diaporthostomataceae* stands out as a particularly rare and understudied fungal group. This rarity may be attributed to their specific habitat requirements, as they thrive in environments undisturbed by adverse conditions. Currently, all known species of *Diaporthostomataceae* have been discovered exclusively in primeval forests or natural reserves.

Species of *Diaporthostomataceae* may act as pathogens on their original hosts, as suggested by symptoms observed during investigations. However, due to their rarity, there is currently no need for active management of the disease, even if they are confirmed as pathogens. Nonetheless, comprehensive pathogenicity tests are required in the future to confirm their role and impact as pathogens.

## Supplementary Material

XML Treatment for Tiania

XML Treatment for Tiania chinensis

XML Treatment for Tiania lithocarpicola

XML Treatment for Tiania quercicola

## ﻿Additional information

### Adherence to national and international regulations

All the fungal strains used in this study have been legally obtained, respecting the Convention on Biological Diversity (Rio Convention).

### Author ORCIDs

Ning Jiang https://orcid.org/0000-0002-9656-8500

Han Xue https://orcid.org/0000-0003-0414-6237

Yong Li https://orcid.org/0000-0002-4406-1329

### Data availability

All of the data that support the findings of this study are available in the main text.

## References

[B1] Alvarez LV Groenewald JZ Crous PW (2016) Revising the *Schizoparmaceae*: *Coniella* and its synonyms *Pilidiella* and *Schizoparme*. Studies in Mycology 85: 1–34. 10.1016/j.simyco.2016.09.001 27766001 PMC5066162

[B2] Barr ME (1978) *Diaporthales* in North America with emphasis on *Gnomonia* and its segregates, Mycologia Memoirs Series, No. 7. Published for the New York Botanical Garden by J. Cramer in collaboration with the Mycological Society of America, Lehre.

[B3] Begoude ADB Gryzenhout M Wingfield MJ Roux J (2010) *Aurifilum*, a new fungal genus in the *Cryphonectriaceae* from *Terminalia* species in Cameroon. Antonie van Leeuwenhoek 98: 263–278. 10.1007/s10482-010-9467-8 20559872

[B4] Braun U Nakashima C Crous PW Groenewald JZ Moreno-Rico O Rooney-Latham S Blomquist CL Haas J Marmolejo J (2018) Phylogeny and taxonomy of the genus *Tubakia* s. lat. Fungal Systematics and Evolution 1(1): 41–99. 10.3114/fuse.2018.01.04 32490362 PMC7259437

[B5] Carbone I Kohn LM (1999) A method for designing primer sets for speciation studies in filamentous ascomycetes. Mycologia 91(3): 553–556. 10.1080/00275514.1999.12061051

[B6] Castlebury LA Rossman AY Jaklitsch WJ Vasilyeva LN (2002) A preliminary overview of the *Diaporthales* based on large subunit nuclear ribosomal DNA sequences. Mycologia 94(6): 1017–1031. 10.1080/15572536.2003.11833157 21156573

[B7] Chadefaud M (1960) Les Végétaux non Vasculaires. Cryptogamie. In: Chadefaud M, Emberger L (Eds) Traité de Botanique Systématique. Masson, Paris vol. 1, 1018 pp.

[B8] Cheewangkoon R Groenewald JZ Summerell BA Hyde KD To-Anun C Crous PW (2009) *Myrtaceae*, a cache of fungal biodiversity. Persoonia 23(1): 55–85. 10.3767/003158509X474752 20198162 PMC2802731

[B9] Cheewangkoon R Groenewald JZ Verkley GJM Hyde KD Wingfield MJ Gryzenhout M Summerell BA Denman S Toanun C Crous PW (2010) Re-evaluation of *Cryptosporiopsiseucalypti* and *Cryptosporiopsis*-like species occurring on *Eucalyptus* leaves. Fungal Diversity 44: 89–105. 10.1007/s13225-010-0041-5

[B10] Crane C Burgess TI (2013) *Luteocirrhusshearii* gen. sp. nov. (*Diaporthales*, *Cryphonectriaceae*) pathogenic to *Proteaceae* in the south Western Australian Floristic Region. IMA Fungus 4(1): 111–122. 10.5598/imafungus.2013.04.01.11 23898417 PMC3719199

[B11] Crous PW Verkley GJ Groenewald JZ (2006) *Eucalyptus* microfungi known from culture. 1. *Cladoriella* and *Fulvoflamma* genera nova, with notes on some other poorly known taxa. Studies in Mycology 55(1): 53–63. 10.3114/sim.55.1.53 18490971 PMC2104730

[B12] Crous PW Summerell BA Alfenas AC Edwards J Pascoe IG Porter IJ Groenewald JZ (2012a) Genera of diaporthalean coelomycetes associated with leaf spots of tree hosts. Persoonia 28(1): 66–75. 10.3767/003158512X642030 23105154 PMC3409416

[B13] Crous PW Summerell BA Shivas RG Carnegie AJ Groenewald JZ (2012b) A reappraisal of Harknessia (Diaporthales), and the introduction of *Harknessiaceae*. Persoonia 28(1): 49–65. 10.3767/003158512X639791 23105153 PMC3409415

[B14] Crous PW Wingfield MJ Guarro J Cheewangkoon R van der Bank M Swart WJ Stchigel AM Cano-Lira JF Roux J Madrid H Damm U Wood AR Shuttleworth LA Hodges CS Munster M de Jesus Yanez-Morales M Zuniga-Estrada L Cruywagen EM de Hoog GS Silvera C Najafzadeh J Davison EM Davison PJ Barrett MD Barrett RL Manamgoda DS Minnis AM Kleczewski NM Flory SL Castlebury LA Clay K Hyde KD Mausse-Sitoe SN Chen S Lechat C Hairaud M Lesage-Meessen L Pawlowska J Wilk M Sliwinska-Wyrzychowska A Metrak M Wrzosek M Pavlic-Zupanc D Maleme HM Slippers B Mac Cormack WP Archuby DI Grunwald NJ Telleria MT Duenas M Martin MP Marincowitz S de Beer ZW Perez CA Gene J Marin-Felix Y Groenewald JZ (2013) Fungal Planet description sheets: 154–213. Persoonia 31(1): 188–296. 10.3767/003158513X675925 24761043 PMC3904050

[B15] Crous PW Carris LM Giraldo A Groenewald JZ Hawksworth DL Hemández-Restrepo M Jaklitsch WM Lebrun M Schumacher RK Stielow JB van der Linde EJ Vilcāne J Voglmayr H Wood AR (2015a) The Genera of Fungi: fixing the application of the type species of generic names – G 2: *Allantophomopsis*, *Latorua*, *Macrodiplodiopsis*, *Macrohilum*, *Milospium*, *Protostegia*, *Pyricularia*, *Robillarda*, *Rotula*, *Septoriella*, *Torula*, and *Wojnowicia*. IMA Fungus 6: 163–198. 10.5598/imafungus.2015.06.01.11 26203422 PMC4500082

[B16] Crous PW Wingfield MJ Guarro J Hernandez-Restrepo M Sutton DA Acharya K Barber PA Boekhout T Dimitrov RA Duenas M Dutta AK Gene J Gouliamova DE Groenewald M Lombard L Morozova OV Sarkar J Smith MT Stchigel AM Wiederhold NP Alexandrova AV Antelmi I Armengol J Barnes I Cano-Lira JF Castaneda RF Contu M Courtecuisse PR da Silveira AL Decock CA de Goes A Edathodu J Ercole E Firmino AC Fourie A Fournier J Furtado EL Geering AD Gershenzon J Giraldo A Gramaje D Hammerbacher A He XL Haryadi D Khemmuk W Kovalenko AE Krawczynski R Laich F Lechat C Lopes UP Madrid H Malysheva EF Marin-Felix Y Martin MP Mostert L Nigro F Pereira OL Picillo B Pinho DB Popov ES Rodas Pelaez CA Rooney-Latham S Sandoval-Denis M Shivas RG Silva V Stoilova-Disheva MM Telleria MT Ullah C Unsicker SB van der Merwe NA Vizzini A Wagner HG Wong PT Wood AR Groenewald JZ (2015b) Fungal Planet description sheets: 320–370. Persoonia 34(1): 167–266. 10.3767/003158515X688433 26240451 PMC4510277

[B17] Crous PW Wingfield MJ Burgess TI Hardy GEStJ Gené J Guarro J Baseia IG García D Gusmão LFP Souza-Mott CM Thangavel R Adamčík S Barili A Barnes CW Bezerra JDP Bordallo JJ Cano-Lira JF de Oliveira RJV Ercole E Hubk V Iturrieta-González I Kubátová A Martín MP Moreau PA Morte A Ordoñez ME Rodríguez A Stchigel AM Vizzini A Abdollahzadeh J Abreu VP Adamčíková K Albuquerque GMR Alexandrova AV Álvarez Duarte E Armstrong-Cho C Banniza S Barbosa RN Bellanger JM Bezerra JL Cabral TS Caboň M Caicedo E Cantillo T Carnegie AJ Carmo LT Castañeda-Ruiz RF Clement CR Čmoková A Conceição LB Cruz RHSF Damm U da Silva BDB da Silva GA da Silva RMF de A Santiago ALCM de Oliveira LF de Souza CAF Déniel F Dima B Dong G Edwards J Félix CR Fournier J Gibertoni TB Hosaka K Iturriaga T Jadan M Jany JL Jurjević Ž Kolařík M Kušan I Landell MF Leite Cordeiro TR Lima DX Loizides M Luo S Machado AR Madrid H Magalhães OMC Marinho P Matočec N Mešić A Miller AN Morozova OV Neves RP Nonaka K Nováková A Oberlies NH Oliveira-Filho JRC Oliveira TGL Papp V Pereira OL Perrone G Peterson SW Pham THG Raja HA Raudabaugh DB Řehulka J Rodríguez-Andrade E Saba M Schauflerová A Shivas RG Simonini G Siqueira JPZ Sousa JO Stajsic V Svetasheva T Tan YP Tkalčec Z Ullah S Valente P Valenzuela-Lopez N Abrinbana M Viana Marques DA Wong PTW Xavier de Lima V Groenewald JZ (2018) Fungal Planet description sheets: 716–784. Persoonia 40: 240–393. 10.3767/persoonia.2018.40.10 30505003 PMC6146637

[B18] Dissanayake AJ Zhu JT Chen YY Maharachchikumbura SS Hyde KD Liu J K (2024) A re-evaluation of *Diaporthe*: refining the boundaries of species and species complexes. Fungal Diversity 126(1): 1–125. 10.1007/s13225-024-00538-7

[B19] Fan X Du Z Liang Y Tian CM (2016) Melanconis (Melanconidaceae) associated with *Betula* spp. in China. Mycological Progress 15: 1–9. 10.1007/s11557-016-1163-2

[B20] Fan XL Bezerra JDP Tian CM Crous PW (2018) Families and genera of diaporthalean fungi associated with canker and dieback of tree hosts. Persoonia 40(1): 119–134. 10.3767/persoonia.2018.40.05 30504998 PMC6146645

[B21] Fan XL Bezerra JDP Tian CM Crous PW (2020) Cytospora (Diaporthales) in China. Persoonia 45(1): 1–45. 10.3767/persoonia.2020.45.01 34456370 PMC8375343

[B22] Gao Y Liu F Cai L (2016) Unravelling *Diaporthe* species associated with *Camellia*. Systematics and Biodiversity 14(1): 102–117. 10.1080/14772000.2015.1101027

[B23] Gryzenhout M Myburg H Wingfield BD (2006) Cryphonectriaceae (Diaporthales), a new family including *Cryphonectria*, *Chrysoporthe*, *Endothia* and allied genera. Mycologia 98: 239–249. 10.1080/15572536.2006.11832696 16894969

[B24] Guterres DC Galvão-Elias S Dos Santos MDM de Souza BCP de Almeida C P Pinho DB Dianese JC (2019) Phylogenetic relationships of *Phaeochorellaparinarii* and recognition of a new family, Phaeochorellaceae (Diaporthales). Mycologia 111(4): 660–675. 10.1080/00275514.2019.1603025 31150307

[B25] Huson DH Bryant D (2024) The SplitsTree App: interactive analysis and visualization using phylogenetic trees and networks. Nature Methods 21(10): 1773–1774. 10.1038/s41592-024-02406-3 39223398

[B26] Jaklitsch WM Voglmayr H (2020) The genus Melanconis (Diaporthales). MycoKeys 63: 69–117. 10.3897/mycokeys.63.49054 32189978 PMC7062851

[B27] Jiang N Li J Piao CG Guo MW Tian CM (2018a) Identification and characterization of chestnut branch-inhabiting melanocratic fungi in China. Mycosphere 9(6): 1268–1289. 10.5943/mycosphere/9/6/14

[B28] Jiang N Voglmayr H Tian CM (2018b) New species and records of *Coryneum* from China. Mycologia 110(6): 1172–1188. 10.1080/00275514.2018.1516969 30481130 PMC6352375

[B29] Jiang N Fan XL Crous PW Tian CM (2019a) Species of *Dendrostoma* (*Erythrogloeaceae*, *Diaporthales*) associated with chestnut and oak canker diseases in China. MycoKeys 48: 67–96. 10.3897/mycokeys.48.31715 30881194 PMC6416227

[B30] Jiang N Fan XL Tian CM (2019b) Identification and pathogenicity of *Cryphonectriaceae* species associated with chestnut canker in China. Plant Pathology 68(6): 1132–1145. 10.1111/ppa.13033

[B31] Jiang N Fan XL Tian CM Crous PW (2020) Reevaluating *Cryphonectriaceae* and allied families in *Diaporthales*. Mycologia 112(2): 267–292. 10.1080/00275514.2019.1698925 32091968

[B32] Jiang N Fan XL Tian CM (2021a) Identification and characterization of leaf-inhabiting fungi from *Castanea* plantations in China. Journal of Fungi 7(10): 64. 10.3390/jof7010064 33477575 PMC7831338

[B33] Jiang N Voglmayr H Bian DR Piao CG Wang SK Li Y (2021b) Morphology and phylogeny of *Gnomoniopsis* (*Gnomoniaceae*, *Diaporthales*) from *Fagaceae* leaves in China. Journal of Fungi 7(10): 792. 10.3390/jof7100792 34682214 PMC8540803

[B34] Jiang N Yang Q Fan XL Tian CM (2021c) *Micromelanconiskaihuiae* gen. et sp. nov., a new diaporthalean fungus from Chinese chestnut branches in southern China. MycoKeys 79: 1–16. 10.3897/mycokeys.79.65221 33958949 PMC8065008

[B35] Katoh K Standley DM (2013) MAFFT multiple sequence alignment software version 7: improvements in performance and usability. Molecular Biology and Evolution 30(4): 772–780. 10.1093/molbev/mst010 23329690 PMC3603318

[B36] Klaubauf S Tharreau D Fournier E Groenewald JZ Crous PW De Vries RP Lebrun MH (2014) Resolving the polyphyletic nature of Pyricularia (Pyriculariaceae). Studies in Mycology 79(1): 85–120. 10.1016/j.simyco.2014.09.004 25492987 PMC4255532

[B37] Lawrence DP Holland LA Nouri MT Travadon R Abramians A Michailides TJ Trouillas FP (2018) Molecular phylogeny of *Cytospora* species associated with canker diseases of fruit and nut crops in California, with the descriptions of ten new species and one new combination. IMA Fungus 9: 333–369. 10.5598/imafungus.2018.09.02.07 30622886 PMC6317586

[B38] Lennox CL Serdani M Groenewald JZ Crous PW (2004) *Prosopidicolamexicana* gen. et sp. nov., causing a new pod disease of *Prosopis* species. Studies in Mycology 50(1): 187–194.

[B39] Liu HY Luo D Huang HL Yang Q (2024) Two new species of *Diaporthe* (*Diaporthaceae*, *Diaporthales*) associated with *Camelliaoleifera* leaf spot disease in Hainan Province, China. MycoKeys 102: 225–243. 10.3897/mycokeys.102.113412 38449924 PMC10915747

[B40] Liu YJ Whelen S Hall BD (1999) Phylogenetic relationships among ascomycetes: evidence from an RNA polymerase II subunit. Molecular Biology and Evolution 16(12): 1799–1808. 10.1093/oxfordjournals.molbev.a026092 10605121

[B41] Luttrell ES (1951) Taxonomy of the *Pyrenomycetes*. University of Missouri Studies, Science Series 24: 1–120.

[B42] Mejía LC Castlebury LA Rossman AY Sogonov MV White JF (2008) Phylogenetic placement and taxonomic review of the genus *Cryptosporella* and its synonyms *Ophiovalsa* and *Winterella* (*Gnomoniaceae*, *Diaporthales*). Mycological Research 112(1): 23–35. 10.1016/j.mycres.2007.03.021 18222674

[B43] Miller MA Pfeiffer W Schwartz T (2010) Creating the CIPRES Science Gateway for inference of large phylogenetic trees. In: Gateway Computing Environments Workshop (GCE), 2010. Institute of Electrical and Electronics Engineers, New Orleans, LA, 1–8. 10.1109/GCE.2010.5676129

[B44] Mu T Lin Y Pu H Keyhani NO Dang Y Lv H Zhao Z Heng Z Wu Z Xiong C Lin L Chen Y Su H Guan X Qiu J (2024) Molecular phylogenetic and estimation of evolutionary divergence and biogeography of the family *Schizoparmaceae* and allied families (*Diaporthales*, *Ascomycota*). Molecular Phylogenetics and Evolution 201: 108211. 10.1016/j.ympev.2024.108211 39368617

[B45] Norphanphoun C Hongsanan S Doilom M Bhat DJ Wen T Senanayake IC Bulgakov TS Hyde KD (2016) *Lamproconiaceae* fam. nov. to accommodate *Lamproconiumdesmazieri*. Phytotaxa 270: 89–102. 10.11646/phytotaxa.270.2.2

[B46] Rambaut A (2018) FigTree v1.4.4, a graphical viewer of phylogenetic trees. https://github.com/rambaut/figtree/releases [Accessed 25 Apr 2020]

[B47] Rayner RW (1970) A mycological colour chart. Commonwealth Mycological Institute, Kew.

[B48] Rehner SA (2001) Primers for elongation factor 1-α (EF1-α). http://ocid.NACSE.ORG/research/deephyphae/EF1primer.pdf

[B49] Ronquist F Huelsenbeck JP (2003) MrBayes 3: Bayesian phylogenetic inference under mixed models. Bioinformatics 19(12): 1572–1574. 10.1093/bioinformatics/btg180 12912839

[B50] Rossman AY Farr DF Castlebury LA (2007) A review of the phylogeny and biology of the *Diaporthales*. Mycoscience 48(3): 135–144. 10.1007/S10267-007-0347-7

[B51] Rossman AY Adams GC Cannon PF Castlebury LA Crous PW Gryzenhout M Jaklitsch WM Mejia LC Stoykov D Udayanga D Voglmayr H Walker DM (2015) Recommendations of generic names in *Diaporthales* competing for protection or use. IMA Fungus 6: 145–154. 10.5598/imafungus.2015.06.01.09 26203420 PMC4500080

[B52] Senanayake IC Crous PW Groenewald JZ Maharachchikumbura SSN Jeewon R Phillips AJL Bhat JD Perera RH Li QR Li WJ Tangthirasunun N Norphanphoun C Karunarathna SC Camporesi E Manawasighe IS Al-Sadi AM Hyde KD (2017) Families of *Diaporthales* based on morphological and phylogenetic evidence. Studies in Mycology 86: 217–296. 10.1016/j.simyco.2017.07.003 28947840 PMC5603113

[B53] Senanayake IC Jeewon R Chomnunti P Wanasinghe IDN Norphanphoun C Karunarathna A Pem D Perera RH Camporesi E McKenzie EHC Hyde KD Karunarathna SC (2018) Taxonomic circumscription of *Diaporthales* based on multigene phylogeny and morphology. Fungal Diversity 93: 241–443. 10.1007/s13225-018-0410-z

[B54] Shuttleworth LA Guest DI (2017) The infection process of chestnut rot, an important disease caused by *Gnomoniopsissmithogilvyi* (*Gnomoniaceae*, *Diaporthales*) in Oceania and Europe. Australasian Plant Pathology 46(5): 397–405. 10.1007/s13313-017-0502-3

[B55] Sogonov MV Castlebury LA Rossman AY White JF (2007) The type species of *Apiognomonia*, *A.veneta*, with its *Discula* anamorph is distinct from *A.errabunda*. Mycological Research 111(6): 693–709. 10.1016/j.mycres.2007.03.013 17604146

[B56] Sogonov MV Castlebury LA Rossman AY Mejía LC White JF (2008) Leaf-inhabiting genera of the *Gnomoniaceae*, *Diaporthales*. Studies in Mycology 62: 1–77. 10.3114/sim.2008.62.01 19287541 PMC2621335

[B57] Sone T Fukiya S Kodama M Tomita F (2000) Molecular structure of rDNA repeat unit in *Magnaporthegrisea*. Bioscience, Biotechnology, and Biochemistry 64(8): 1733–1736. 10.1271/bbb.64.1733 10993165

[B58] Stamatakis A Hoover P Rougemont J (2008) A rapid bootstrap algorithm for the RAxML web servers. Systematic Biology 57(5): 758–771. 10.1080/10635150802429642 18853362

[B59] Suwannarach N Kumla J Sri-Ngernyuang K Lumyong S (2016) A new endophytic fungus, *Chrysofoliabarringtoniae* sp. nov., from Thailand. Mycoscience 57(5): 361–365. 10.1016/j.myc.2016.06.003

[B60] Tennakoon DS Kuo CH Maharachchikumbura SS Thambugala KM Gentekaki E Phillips AJ Bhat DJ Wanasinghe DN de Silva NI Promputtha I Hyde KD (2021) Taxonomic and phylogenetic contributions to *Celtisformosana*, *Ficusampelas*, *F.septica*, *Macarangatanarius* and *Morusaustralis* leaf litter inhabiting microfungi. Fungal Diversity 108(1): 1–215. 10.1007/s13225-021-00474-w

[B61] Udayanga D Miriyagalla SD Manamgoda DS Lewers KS Gardiennet A Castlebury LA (2021) Molecular reassessment of diaporthalean fungi associated with strawberry, including the leaf blight fungus, *Paraphomopsisobscurans* gen. et comb. nov. (*Melanconiellaceae*). IMA Fungus 12(1): 15. 10.1186/s43008-021-00069-9 34158123 PMC8218473

[B62] Vilgalys R Hester M (1990) Rapid genetic identification and mapping of enzymatically amplified ribosomal DNA from several *Cryptococcus* species. Journal of Bacteriology 172(8): 4238–4246. 10.1128/jb.172.8.4238-4246.1990 2376561 PMC213247

[B63] Voglmayr H Jaklitsch WM (2008) *Prosthecium* species with *Stegonsporium* anamorphs on *Acer*. Mycological Research 112(8): 885–905. 10.1016/j.mycres.2008.01.020 18554889

[B64] Voglmayr H Jaklitsch WM (2014) *Stilbosporaceae* resurrected: generic reclassification and speciation. Persoonia 33: 61–82. 10.3767/003158514X684212 25737594 PMC4312938

[B65] Voglmayr H Rossman AY Castlebury LA Jaklitsch WM (2012) Multigene phylogeny and taxonomy of the genus Melanconiella (Diaporthales). Fungal Diversity 57: 1–44. 10.1007/s13225-012-0175-8

[B66] Voglmayr H Castlebury LA Jaklitsch WM (2017) *Juglanconis* gen. nov. on *Juglandaceae*, and the new family Juglanconidaceae (Diaporthales). Persoonia 38(1): 136–155. 10.3767/003158517X694768 29151630 PMC5645181

[B67] Walker DM Castlebury LA Rossman AY Sogonov MV White JF (2010) Systematics of genus *Gnomoniopsis* (*Gnomoniaceae*, *Diaporthales*) based on a three gene phylogeny, host associations and morphology. Mycologia 102(6): 1479–1496. 10.3852/10-002 20943552

[B68] Wehmeyer LE (1975) The pyrenomycetous *Fungi*. Mycologia Memoirs 6: 1–250.

[B69] White TJ Bruns T Lee SJWT Taylor JW (1990) Amplification and direct sequencing of fungal ribosomal RNA genes for phylogenetics. PCR protocols: a guide to methods and applications 18: 315–322. 10.1016/B978-0-12-372180-8.50042-1

[B70] Xavier KV Kc AN Crous PW Groenewald JZ Vallad GE (2019) *Dwiroopapunicae* sp. nov.(*Dwiroopaceae* fam. nov., *Diaporthales*), associated with leaf spot and fruit rot of pomegranate (*Punicagranatum*). Fungal Systematics and Evolution 4(1): 33–41. 10.3114/fuse.2019.04.04 32467905 PMC7241677

[B71] Yang Q Fan XL Crous PW Liang YM Tian CM (2015) *Cytospora* from *Ulmuspumila* in northern China. Mycological Progress 14: 1–12. 10.1007/s11557-015-1096-1

[B72] Yang Q Fan XL Du Z Tian CM (2017) *Diaporthosporellaceae*, a novel family of *Diaporthales* (*Sordariomycetes*, *Ascomycota*). Mycoscience 59(3): 229–235. 10.1016/j.myc.2017.11.005

[B73] Zhang N Blackwell M (2001) Molecular phylogeny of dogwood anthracnose fungus (*Disculadestructiva*) and the *Diaporthales*. Mycologia 93(2): 355–365. 10.1080/00275514.2001.12063167

[B74] Zhang Z Liu X Tao M Liu X Xia J Zhang X Meng Z (2023) Taxonomy, phylogeny, divergence time estimation, and biogeography of the family *Pseudoplagiostomataceae* (*Ascomycota*, *Diaporthales*). Journal of Fungi 9(1): 82. 10.3390/jof9010082 36675903 PMC9860658

[B75] Zhu Y Ma L Xue H Li Y Jiang N (2024) New species of *Diaporthe* (*Diaporthaceae*, *Diaporthales*) from *Bauhiniavariegata* in China. MycoKeys 108: 317–335. 10.3897/mycokeys.108.128983 39310741 PMC11415621

